# Antibody targeting of claudin-1 as a potential colorectal cancer therapy

**DOI:** 10.1186/s13046-017-0558-5

**Published:** 2017-06-28

**Authors:** S. Cherradi, A. Ayrolles-Torro, N. Vezzo-Vié, N. Gueguinou, V. Denis, E. Combes, F. Boissière, M. Busson, L. Canterel-Thouennon, C. Mollevi, M. Pugnière, F. Bibeau, M. Ychou, P. Martineau, C. Gongora, M. Del Rio

**Affiliations:** 1 Institut de Recherche en Cancérologie de Montpellier (IRCM), Inserm U1194, Université de Montpellier, Institut Régional du Cancer de Montpellier (ICM), 208 rue des Apothicaires, F-34298 Montpellier Cedex 5, France; 2 Institut régional du Cancer de Montpellier (ICM), Montpellier, F-34298 France

**Keywords:** Metastatic, Colorectal cancer, Claudin-1, Antibody, Targeted therapy

## Abstract

**Background:**

Metastatic colorectal cancer (mCRC) is one of the major causes of cancer-related death. Despite the substantial progress in mCRC management, it remains important to identify new therapeutic options and biological markers for personalized medicine. Here, we investigated the expression of claudin-1 (CLDN1), a major tight junction transmembrane protein, in the different colorectal cancer (CRC) molecular subtypes and then assessed the anti-tumor effect of a new anti-CLDN1 monoclonal antibody (mAb).

**Methods:**

Gene expression profiling and immunochemistry analysis of normal and tumor tissue samples from patients with stage IV CRC were used to determine *CLDN1* gene expression. Then, the 6F6 mAb against CLDN1 extracellular part was generated. Its effect on CRC cell cycle, proliferation, survival and migration was assessed in vitro, using a 3D cell culture system, flow cytometry, clonogenic and migration assays. In vivo, 6 F6 mAb efficacy was evaluated in nude mice after subcutaneous xenografts or intrasplenic injection of CRC cells.

**Results:**

Compared with normal mucosa where it was almost exclusively cytoplasmic, in CRC samples *CLDN1* was overexpressed (*p* < 0.001) and mainly localized at the membrane. Moreover, it was differentially expressed in the various CRC molecular subtypes. The strongest expressions were found in the consensus molecular subtype CMS2 (*p* < 0.001), the transit-ampliflying (*p* < 0.001) and the C5 subtypes (*p* < 0.001). Lower *CLDN1* expression predicted a better outcome in the molecular subtypes C3 and C5 (*p* = 0.012 and *p* = 0.004, respectively). CLDN1 targeting with the 6 F6 mAb led to reduction of survival, growth and migration of CLDN1-positive cells. In preclinical mouse models, the 6F6 mAb decreased tumor growth and liver metastasis formation.

**Conclusion:**

Our data indicate that CLDN1 targeting with an anti-CLDN1 mAb results in decreased growth and survival of CRC cells. This suggests that CLDN1 could be a new potential therapeutic target.

**Electronic supplementary material:**

The online version of this article (doi:10.1186/s13046-017-0558-5) contains supplementary material, which is available to authorized users.

## Background

Colorectal cancer (CRC) is one of the major causes of cancer-related death in the Western world. When localized, CRC is often curable by surgery, but the prognosis of patients with metastatic CRC (mCRC) remains very poor [1]. Standard chemotherapy regimens (FOLFIRI or FOLFOX) used in combination with targeted therapies have improved response rates and survival [2–4]. However, 30 to 50% of patients are intrinsically resistant to treatments and nearly all patients who are initially responsive will eventually develop resistance. Therefore, other first-line treatment options for patients with mCRC are required.

Claudins (CLDN) are integral membrane proteins that determine the barrier features of tight junctions [5, 6] and are considered potential therapeutic targets for antibody-based treatments [7]. They have four transmembrane domains, two extracellular loops and cytoplasmic tails [8, 9]. Claudin-1 (CLDN1) is one of the 27 members of the claudin family [10]. Several groups have reported increased expression of CLDN1 in primary CRC and metastases as well as in CRC cell lines but with marked differences in its localization [11–14]. Indeed, immunohistochemistry analyses in CRC and normal mucosa samples showed that cancer cells display a membranous [11] or membranous/cytoskeletal [12] staining of CLDN1. Other studies found a strong CLDN1 expression at the cell–cell boundaries and in the cytoplasm of cancer cells [13]. Moreover, CLDN1 nuclear localization in tumor samples was reported [14]. Aberrant CLDN1 expression in tumor cells can lead to alteration of the tight junction structure and function, or dysregulation of cell signaling pathways [15]. CLDN1 could be involved in WNT and NOTCH signaling. Indeed, CLDN1 is a known target of TCF/LEF signaling [13], but seems also to participate in the regulation of the WNT signaling pathway [16]. Through NOTCH signaling upregulation, CLDN1 has a role in colon epithelium homeostasis [17] and in promoting colon tumorigenesis [18].

Much effort has been focused on understanding CLDN1 function and regulation in cancer; however, little is known about its potential usefulness as a therapeutic target. Therefore, the aim of this study was to accurately assess CLDN1 expression in CRC samples and to determine the potential of CLDN1 as a therapeutic target for antibody-based treatments in CRC.

## Methods

### CRC samples

For gene expression profiling, we selected 143 tumor samples from 143 patients included in three cohorts: the prospective single-center study REGP (19 patients) (GSE62322) [19, 20], the retrospective multi-center study COSIVAL (68 patients) and the prospective multi-center study BIOCOLON (56 patients) (GSE62080 and GSE72970) [21]. For these three studies, Inclusion criteria were: histologically proven colon adenocarcinoma, advanced and bidimensionally measurable tumor (stage IV), age between 18 and 75 years, and World Health Organization (WHO) performance status ≤2. Before any treatment, all patients underwent surgery for primary tumor resection or endoscopic biopsy.

For western blot analysis, 13 additional tumor samples from the prospective single-center study REGP but not included in the 143 samples were used.

For immunohistochemistry analysis, tissue samples from 52 additional patients with CRC were retrospectively selected from the Institute of Cancer Research of Montpellier pathology files only when normal mucosa, adenoma and adenocarcinoma samples were available for the same patient.

All the studies using human tissue samples were approved by the relevant ethics committees and all participants were informed about the study objectives and methods and signed a written informed consent before enrolment.

### Gene expression analysis

Colon samples (normal colon, primary tumor and hepatic metastasis samples for the REG/P study, but only primary tumor specimens for the COSIVAL and BIOCOLON trials) were collected at the time of surgery following a standardized procedure to obtain high quality RNA [22]. Samples were then hybridized to human genome U133 Plus 2.0 arrays (Affymetrix Inc., Santa Clara, CA).

To identify new therapeutic targets for antibody-based therapy in mCRC, we compared the gene expression profiles of normal mucosa (*n* = 17), primary tumor (*n* = 20) and hepatic metastases (*n* = 19) tissue samples.

As CRC heterogeneity must be taken into account when comparing gene expression profiles, primary tumor samples (*n* = 143) were classified using the CRC molecular classifications based on gene expression profiles that have been proposed by three independent groups [23–25] and the recent consensus classification [26]. Briefly, De Sousa E. Melo et al. proposed to group tumors in three classes: CCS1 (CRC with microsatellite instability, MSI), CCS2 (cancer with chromosomal instability, CIN) and CCS3 (new subtype) [25]. Sadanandam et al. identified five molecular subtypes, based on the cell phenotype: Goblet-like, Transit-Amplifying (TA), Enterocyte, Stem-like, and Inflammatory [24]. Marisa et al. described six molecular subtypes (C1 to C6) with the following main features: C1 = CIN and downregulation of immune pathways, C2 = MSI, C3 = mutated KRAS, C4 = stem cell phenotype-like, C5 = CIN and upregulation of the WNT pathways, and C6 = CIN and normal-like gene expression profile [23]. Finally, starting from six previously published signatures, an international consortium presented a classification with four consensus subtypes: MSI (CMS1), canonical (CMS2), metabolic (CMS3), and mesenchymal (CMS4) [26] (for review [27]). The CRC sample distribution according to the molecular subtype is shown in Additional file 1: Table S1.

### Immunohistochemistry analysis

Samples were assembled in a tissue micro-array (TMA) using three tissue cores (0.6-mm diameter each) as previously described [28]. Briefly, 3-μm sections of the TMA were de-paraffinized and rehydrated in graded alcohols. Heat-induced antigen retrieval was performed by incubating TMA sections in EDTA buffer (pH 9) at 98 °C in a water bath for 20 min. After neutralization of the endogenous peroxidase activity, TMA sections were incubated with a polyclonal anti-CLDN1 antibody (JAY-8, Zymed laboratories Inc, CA, USA) or antibody diluent (Dako, Glostrup, Denmark) alone for 60 min. Primary antibody binding was visualized using the Envision® system and the Dako Autostainer® (Dako, Glostrup, Denmark). The percentage of CLDN1-positive cells and the staining intensity (0, no staining; 1, yellowish; 2, brown; and 3, dark brown) were evaluated for each individual TMA spot.

### Western blot analysis

Tumor tissue samples from patients were directly grinded in lysis buffer (150 mM NaCl, 10 mM Tris pH 7.4, 1 mM EDTA, 1 mM EGTA, 1% SDS, 1% Triton X-100, 0.5% NP-40, 2 mM PMSF, 100 mM NaF, 10 mM sodium orthovanadate, one cocktail protease inhibitor tablet for 10 ml) using a Mixer Mill® MM 300 unit (Qiagen, Valencia, CA). Protein concentration was determined with the Bradford assay (Pierce Coomassie Plus Protein Assay). Then, 50 μg of total proteins were resolved by 12% SDS-PAGE and transferred onto nitrocellulose membranes (Whatman® *Protran*®, pore size 0.45 μm). Non-specific binding sites were blocked with 5% (wt/vol) nonfat milk in PBS with 0.1% (vol/vol) Tween 20 (PBS-T) at room temperature for 1 h and then membranes were incubated at 4 °C with a polyclonal anti-CLDN1 antibody (JAY-8) overnight. Membranes were then washed and incubated with the appropriate horseradish peroxidase-conjugated secondary antibody for 1 h. Revelation was performed with a chemiluminescence system (Amersham Biosciences); β-tubulin expression was used for normalization.

### Subcellular protein extraction

Protein extraction was carried out as previously described [29]. For each sample, 20-μm thick sections were cut with a cryotome, mixed in liquid nitrogen and gently ground with a micro-pestle. For subcellular protein extraction, the ProteoExtract Subcellular Proteome Extraction Kit was used according to the manufacturer’s instructions (Calbiochem). Subcellular fractions (10 μg/each) were loaded on 12% SDS-PAGE gels. Immunoblotting was done as described above with the following primary antibodies: anti-CLDN1 (JAY-8), -CD71 (Invitrogen), -Histone H3 (Pierce) and -β-tubulin (Sigma T4026).

### Cell lines

The following human CRC cell lines were used: SW480 (ATCC CCL-228), SW620 (ATCC CCL-227), Caco-2 (ATCC HTB-37), Difi [30] (a gift from C. Montagut, Department of Medical Oncology, Hospital del Mar, Barcelona, Spain), HCT116 (CCL-247), and LS174T (ATCC CL-188). To obtain the CLDN1-positive SW480 cell line (SW480-CLDN1), SW480 cells were stably transfected with the human CLDN1 cDNA clone (Invitrogen MGC collection) or with empty vector (pcDNA) using the jetPRIME™ transfection reagent (Polyplus-transfection Inc., France). CLDN1-positive clones were selected by growing transfected cells in the presence of 500 μg/ml of geneticin. For CLDN1 silencing, SW620 was transduced with the pSIREN vector containing the shRNA against CLDN1 (SW620shCLDN1) or against luciferase (shLUC, negative control). After 24 h, cells were selected with 1 μg/mL puromycin and stable clones were pooled. All transient transfections were done using the jetPRIME™ transfection reagent.

### Production of anti-CLDN1 mAb 6 F6

For antibody production, 6–8 weeks-old female BALB/c mice (Harlan, Gannat, France) were challenged by intra-peritoneal (i.p.) injection of 4 million mouse NIH cells transiently transfected with CLDN1 cDNA (NIH-CLDN1 cells) every two weeks (five injections in total). NIH-CLDN1 cells were mixed with complete Freund’s adjuvant (Sigma) for the first injection, and with incomplete Freund’s adjuvant (Sigma) for the other four injections. An intravenous booster injection of NIH-CLDN1 cells was given three months after the fifth immunization. Three days later, spleen cells from immunized mice were fused with the mouse myeloma cell line P3-X63-Ag.8.653 to produce mouse hybridomas. Supernatants from newly generated clones were screened by fluorescence-activated cell sorting (FACS) using SW480-CLDN1 and SW480 cells (negative control). The screening results were confirmed by performing and additional screening using SW620 and SW620-shCLDN1 cells. The anti-CLDN1 hybridoma 6 F6 clone was selected and cloned by limiting dilution. Antibody isotyping showed that 6 F6 was an IgG3k.

All animal experiments were performed in compliance with the French government guidelines for experimental animal studies (agreement CEEA-LR-12052).

### Radiolabeling and SPECT-CT imaging

Female athymic nude mice (6–8-week-old) were purchased from Harlan. The 6 F6 mAb was radiolabeled with ^125^I (Perkin Elmer) at the specific activity of 370 MBq/mg for single-photon emission computed tomography (SPECT) imaging, using the IODO-GEN (Pierce Chemical Co.) method. After tail vein injection of 16 MBq/50 μg ^125^I-labeled 6 F6, whole-body single photon emission tomography/computed tomography (SPECT/CT) images were acquired with a 4-head multiplexing multi-pinhole NanoSPECT camera (Bioscan Inc., Washington, USA) at various times (48, 72 and 96 h). Concomitantly, whole-body micro-CT images were acquired for anatomic co-registration with SPECT data. Reconstructed SPECT and CT data were visualized and co-registered using Invivoscope®.

### Clonogenic assay

Colorectal cancer cells were seeded in a 6-well plate (150, 250 or 400 cells/well) and allowed to adhere at 37 °C overnight. Then, 1 ml of RPMI with or without 6 F6 mAb (final concentration: 100 μg/ml) was added to each well and cells were cultured for six days. After six more days in medium without antibody, plates were read using a Celigo™ imaging cytometer and the “Single colony verification” application. The Celigo™ cytometer is a benchtop *in situ* cellular analysis system that provides images of wells using bright-field illumination (Nexcelom Bioscience, MA, USA).

### Establishment of three-dimensional (3D) spheroid cultures

Ultra-low attachment, round-bottomed 96-well plates (Corning Costar) were used for spheroid formation. SW480, SW480-CLDN1 or SW620 cells were seeded at a density of 5 × 10^4^. Cells aggregated and merged in 3D spheroids within 24–72 h. Images of wells were taken with a phase-contrast microscope using a 5× objective or captured with the Celigo™ imaging cytometer using the “Tumorosphere” application. Cell viability was assessed with the CellTiter-Glo Luminescent Cell Viability Assay (Promega, Madison, WI, USA). After addition of 100 μl of CellTiter Glo reagent to each well for 10 min, luminescence was measured on a 1450 MicroBeta TriLux Luminescence microplate reader (Perkin Elmer).

### Cell cycle and proliferation analysis in spheroids

Spheroids were prepared by plating 1000 DiFi cells per well in ultra-low attachment 96-well plates, and growing them in the presence of 100 μg/ml of the 6 F6 mAb or irrelevant mAb (retuximab) for 5 days. For cell cycle analysis, cells were pelleted, trypsinized, washed with PBS, fixed in 75% ethanol, and stained with 40 *μ*g/ml propidium iodide in the presence of 100 *μ*g ml^−1^ RNAse (Qiagen). The cell cycle distribution was determined with a FC500 Beckman Coulter Flow Cytometer using the FL-3 channel. Cells were gated on a dot plot that displayed the DNA-pulse-peak *vs* the DNA-pulse area to exclude doublets. Cell cycle distributions were illustrated using the Flow Jo analysis software (Treestar, FLOWJO, Ashland, OR, USA).

At day 4 of culture, cell proliferation was measured by incubating cells with 5-ethynyl-2′-deoxyuridine (EdU) for 24 h. EdU is incorporated into DNA during active DNA synthesis. Then, after cell trypsinization and fixation/permeabilization in 75% ethanol/PBS, incorporated EdU was labeled and detected with the Click-iT EdU Alexa Fluor 488 Flow Cytometry Assay Kit (Invitrogen). Cells were then incubated with 1 μg/ml of 4′,6-diamidino-2-phenylindole (DAPI) in PBS/0.1% Triton X100 at 37 °C for 30 min. The Celigo™ “Expression Analysis” (Target 1 + Mask) application was used to quantify the fluorescent signal and for data analysis. Cells were identified using the DAPI nuclear stain and DNA synthesis was quantified by measuring EdU incorporation.

### Mouse xenograft models

1.5 × 10^6^ SW620 cells or 3 × 10^6^ DiFi cells were suspended in culture medium and injected subcutaneously (s.c.) into the right flank of 6–8-week-old female athymic nude mice from Harlan. When the tumor volume reached approximately 100 mm^3^, mice were randomized in different groups and treated by i.p. injection of 0.9% NaCl or 6F6 mAb (15 mg/Kg per injection) twice a week for three consecutive weeks for the first experiment and thrice a week for the second experiment. Tumors were measured bi-weekly with a caliper and volumes calculated with the formula: D1 x D2 x D3/2.

### Intrasplenic hepatic colonization model

In each experiment, 2 million luciferase-expressing SW620 cells (SW620-LUC cells) were injected in the spleen of 6–8-week-old female athymic nude mice. Spleen was removed 2 min after cell injection. On day 1 after injection, mice were randomly divided in two groups of 10 mice that were treated either with 15 mg/kg of 6 F6 mAb or 0.9% NaCl by i.p. injection, thrice per week. To evaluate metastatic formation and dissemination, luciferase expression was monitored by luminescence imaging after injection of luciferin (Camera Ivis Lumina II, PerkinElmer®) once per week. At week 5 after surgery, mice were sacrificed, livers were removed, photographed and macroscopically visible metastases on the liver surface were counted.

### Statistical analysis

Statistical analysis was done using the STATA 13.0 software (StataCorp). For gene expression or immunohistochemistry experiments, differences between groups were analyzed using the Kruskall Wallis/Dunn’s test. Correlations between *CLDN1* gene expression and progression-free survival (PFS) and overall survival (OS) were evaluated in the entire group (*n* = 143 patients) and according to the tumor molecular subtype. To this end, the 143 patients were divided in two groups based on the median *CLDN1* gene expression (i.e., 9.75 [arbitrary units]). PFS and OS values were compared using the Kaplan-Meier method and differences between survival distributions assessed using the log-rank test.

The paired *t*-test was used to compare the effect of incubation with the 6 F6 mAb in in vitro experiments.

In in vivo experiments, a linear mixed regression model was used to determine the relationship between tumor growth and number of days after injection. The fixed part of the model included variables corresponding to the number of post-graft days and different treatment groups. Interaction terms were built into the model; random intercepts and random slopes were included to take into account the time effect. The coefficients of the model were estimated by maximum likelihood. Survival rates were estimated from the date of the injection until the date when the tumor reached a volume of 1500 mm^3^ using the Kaplan–Meier method. Survival curves were compared using the log-rank test. For the hepatic colonization experiments, differences between groups were evaluated with the Mann-Whitney *U* test. For all experiments, differences were considered to be significant when *P* < 0.05.

## Results

### CLDN1 is overexpressed at the membrane of CRC cells

Gene expression analysis of CRC samples showed that *CLDN1* was overexpressed in primary tumors (*p* < 0.0001) and metastases (*p* < 0.0001) compared with normal mucosa (Additional file 2: Figure S1A). In 15 pairs of normal mucosa and primary tumors, *CLDN1* expression in tumors was 2 to 27 times higher than in the matched normal mucosa (Additional file 2: Figure S1B).

**Fig. 1:**
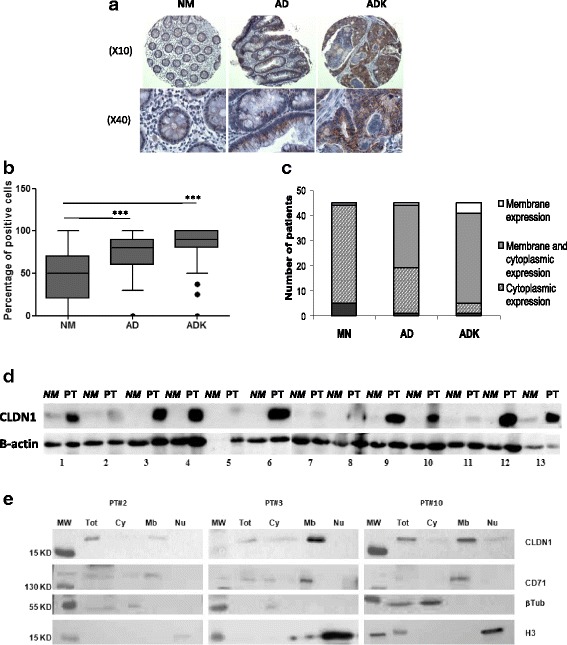
CLDN1 overexpression in CRC tissue samples. **a** CLDN1 staining by immunochemistry in normal mucosa (NM), adenoma (AD) and adenocarcinoma (ADK) samples from the same patient. **b** Percentage of CLDN1-positive cells relative to all cells in the TMA spot in paired NM, AD and ADK samples from 45 patients with CRC; *** = *p* < 0.0001, Kruskall Wallis/Dunn’s test. **c** CLDN1 localization in NM, AD and ADK samples from 45 patients with CRC. **d** Western blot analysis of CLDN1 expression in 13 matched tissue samples. NM = normal mucosa; PT = primary tumor. **e** Subcellular fractionation of three primary tumor samples. Cy = cytoplasm, Mb = membrane, Nu = nucleus, Tot = total extract. Anti-β-tubulin, -CD71 and -histone H3 antibodies were used as subcellular markers

Among the 52 matched samples, 45 samples with matched normal tissue, adenoma and adenocarcinoma were used to confirm CLDN1 overexpression by immunohistochemistry (Fig. 1a). No staining was observed in the sections incubated with diluent alone. CLDN1 staining was significantly higher in the adenoma and adenocarcinoma samples compared with the matched normal mucosa (*p* < 0.001) (Fig. 1b). CLDN1 was expressed in the cytoplasm in 87% of normal mucosa (39/45 patients) and in 40% of adenoma samples (18/45). Conversely, it was localized both at the membrane and in the cytoplasm in 56% of adenoma (25/45) and 80% of adenocarcinoma samples (36/45). In 9% of adenocarcinoma samples (4/45), it was exclusively localized at the cell membrane (Fig. 1c).

CLDN1 overexpression in CRC tumors was also assessed by western blotting of matched samples from 13 additional patients (Fig. 1d). CLDN1 was strongly overexpressed in eight of these primary CRC tumors, compared with the matched normal mucosa, and moderately in three others. Western blot analysis of subcellular protein extracts from three of the primary tumor samples with moderate or strong CLDN1 expression showed that CLDN1 expression was mainly localized at the membrane (Fig. 1e).

### CLDN1 is differently expressed in CRC molecular subtypes and has a prognostic value

**Fig. 2:**
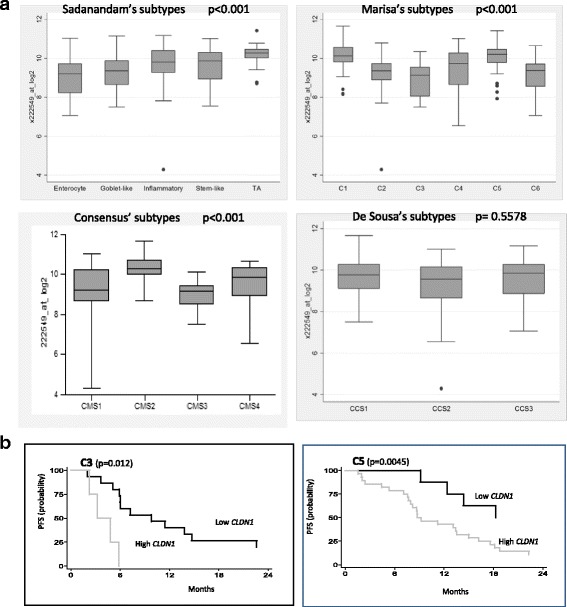
Differential expression and clinical value of CLDN1 gene expression in CRC samples from patients with mCRC. **a** CLDN1 gene expression in 143 primary CRC samples classified according to the five molecular subtypes described by Sadanandam et al. [24], the six molecular subtypes described by Marisa et al. [23], the three molecular subtypes described by De Sousa et al. [25], and the four consensus subtypes [26] (Kruskall Wallis/Dunn’s test). **b** Associations between CLDN1 gene expression level and progression-free survival (PFS) in patients with C3 and C5 subtype tumors (log-rank test). High CLDN1: >median; low CLDN1: <median CLDN1 expression (9.75 arbitrary units)

Then, *CLDN1* gene expression was evaluated in 143 primary tumor samples that were classified in the different molecular subtypes [21]. *CLDN1* was significantly up-regulated in the TA subtype of the Sadanandam’s classification compared with the other subtypes (*p* < 0.001) (Fig. 2a). Among the Marisa’s subtypes, the strongest *CLDN1* expression was found in the C1 and C5 subtypes (*p* < 0.001) (Fig. 2a). *CLDN1* expression was slightly increased in CCS1 compared with CSS2 and CSS3 (De Sousa E Melo’s subtypes) (Fig. 2a). *CLDN1* differential overexpression was confirmed in the four molecular subtypes of the consensus classification [26]. Specifically, *CLDN1* expression was higher in the CMS2 consensus subtype that includes the TA, C1, C5 and CCS1 subtypes (Fig. 2b).

No significant correlation was found between *CLDN1* gene expression (low/high) and OS and PFS in the whole patients’ group. Conversely, PFS was significantly longer in patients with C3 and C5 tumors with low *CLDN1* gene expression. Indeed, the median PFS values were 9.8 and 3.2 months for patients with C3 tumors with low or high *CLDN1* gene expression, respectively (*p* = 0.012), and 18 and 8.8 months for patients with C5 tumors with low or high *CLDN1* gene expression, respectively (*p* = 0.0045) (Fig. 2c).

### Characterization of a monoclonal antibody against human CLDN1

**Fig. 3:**
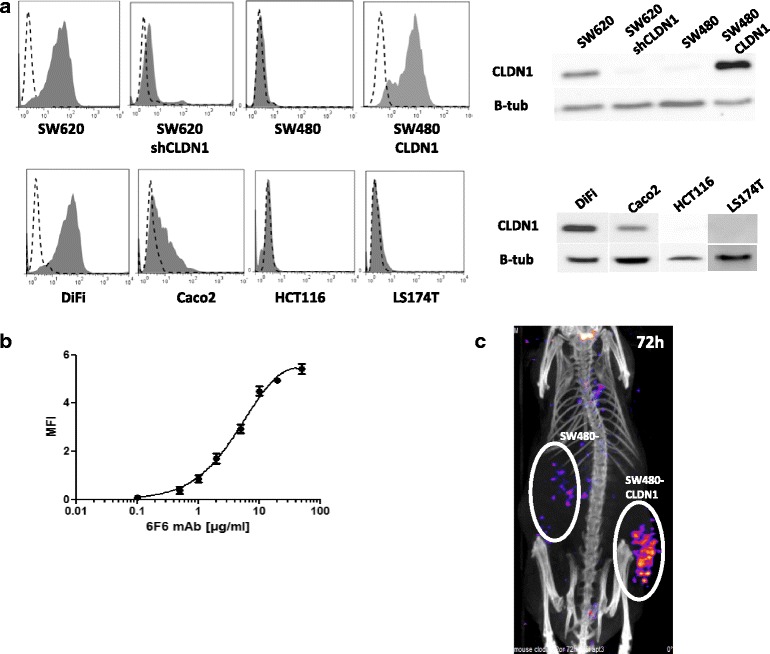
Specificity and affinity of the anti-CLDN1 mAb 6F6. **a** Reactivity of 10 μg/ml of purified 6F6 mAb towards different CRC cell lines that express or not CLDN1, determined by FACS analysis (Additional file 3). *Left*: FACS histograms of cells incubated with (*gray*) or not (*dotted line*) the 6F6 mAb; *Right*: quantification of CLDN1 expression by western blotting using the anti-CLDN1 polyclonal antibody JAY8. **b** Determination of the half saturation binding considered as the apparent Kd. SW620 cells were incubated with increasing concentrations of the 6F6 mAb and binding was assessed by FACS **c** Biodistribution of ^125^I-labeled 6F6. Images were acquired three days after intravenous injection of 500 μCi of ^125^I-6F6 mAb in the tail vein of mice bearing SW480 or SW480-CLDN1 cell xenografts

A murine mAb against the extracellular part of human CLDN1 (6 F6 mAb) was generated. Its specificity was demonstrated by showing that (i) the 6 F6 mAb bound only to CLDN1-positive CRC cell lines (SW620, SW480-CLDN1, Difi, Caco2), but not to CLDN1-negative cell lines (SW480, SW620shCLDN1, HCT116, LS174T) (Additional file 2: Figure S2A and Fig. 3a), (ii) the 6 F6 mAb could bind to non-permeabilized SW480-CLDN1 cells, but not to parental SW480 cells (Additional file 2: Figure S2B; Additional file 3) and (iii), the 6 F6 mAb, but not an irrelevant mAb could bind to SW620 membrane extracts, as indicated by Surface Plasmon Resonance (Additional file 2: Figure S2C; Additional file 3). The 6 F6 mAb bound to SW620 cells with an apparent K_D_ of 37 nM ± 7.8 (Fig. 3b). Then, 6 F6 cross-reactivity with murine CLDN1, human CLDN7 and human CLDN8 was assessed after transient transfection of human CLDN8 and murine or human CLDN1 in SW480 cells that express CLDN7 [31] (Additional file 2: Figure S2D, upper panels). The 6 F6 mAb did not cross-react with CLDN8, CLDN7 and mouse CLDN1. (Additional file 2: Figure S2D, lower panels). Finally, to determine the tumor uptake and the ability of the 6 F6 mAb to specifically target CLDN1 in vivo, ^125^I-labeled 6 F6 was injected in mice with SW480-CLDN1 and SW480 cell xenografts. Analysis of the SPECT/CT data showed high and specific uptake of ^125^I labeled-6F6 mAb only in SW480-CLDN1 tumor cell xenografts (Fig. 3c).

### CLDN1 targeting reduces CRC cell survival, growth, proliferation and migration

**Fig. 4:**
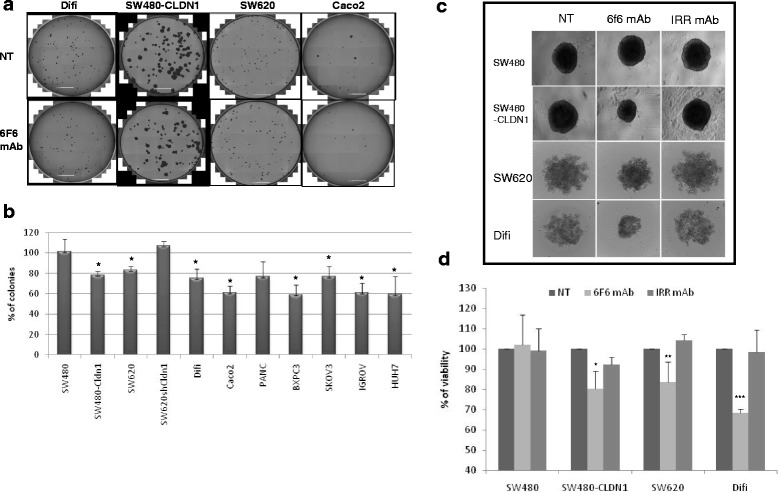
In vitro effects of the 6F6 mAb on CRC cell survival and growth. **a** Clonogenic assay in CLDN1-positive CRC cells in the presence or not (NT) of 100 μg/ml of the 6 F6 mAb. Images were obtained using a Celigo™ imaging cytometer. Scale bar: 5 mm. **b** Quantification of the clonogenic assay results: the histogram shows the percentage of colonies in treated cultures (i.e., the ratio between the number of colonies in the treated well and the number of colonies in the untreated well × 100); * = *p* < 0.05 (paired *t*-test). **c** Effect of the 6F6 mAb on growth of 3D spheroids. Cells were incubated or not (NT) with 100 μg/ml of 6F6 or irrelevant (IRR) mAb. Representative images of spheroids after 72 h of culture on Ultra-low attachment plates. **d** Bioluminescence cytotoxicity assay to determine cell viability in spheroids grown in the presence or not (NT) of the 6F6 or an irrelevant (IRR) mAb. Cell viability was assessed by measuring the ATP content. Results are shown as the ratio between the ATP content in treated spheroids and the ATP content in untreated spheroids × 100; * = *p* < 0.05; ** = *p* < 0.01; *** = *p* < 0.001 (paired *t*-test)

The number of colony-forming cells was significantly reduced in CLDN1-positive CRC cell lines incubated with the 6 F6 mAb, compared with untreated cells (Fig. 4a, b). To confirm that this effect was specifically caused by CLDN1 targeting, the same experiment was performed using two CLDN1-negative cell lines (SW480 and SW620shCLDN1) (Fig. 4b). In these cells, the number of colony-forming cells was not affected by incubation with 6F6 compared with untreated cells. To further validate these results, the 6 F6 mAb was tested in other cancer cell lines that overexpress CLDN1 (BXPC3, PANC-1, SKOV-3, IGROV1 and HuH-7 cells) (Additional file 2: Figure S3 and Fig. 4b). The 6F6 mAb inhibited colony formation (17 to 41% of inhibition) in all tested CLDN1-positive cell lines.

In spheroids made of CLDN1-positive CRC cell lines (SW480-CLDN1, SW620 and Difi cells), spheroid size was smaller in antibody-treated than in untreated cultures (Fig. 4c). Conversely, no effect was observed in spheroids incubated with an irrelevant antibody or in spheroids made of CLDN1-negative SW480 cells. Similarly, the viable cell number (between 17 and 32%) was significantly reduced in spheroids made of SW480-CLDN1 (*p* = 0.03), SW620 (*p* = 0.005) and Difi (*p* = 0.0001) cells incubated with the 6F6 mAb, but not in SW480 spheroids (*p* = 0.7), compared with controls (untreated cultures or incubated with the irrelevant antibody) (Fig. 4d).

**Fig. 5:**
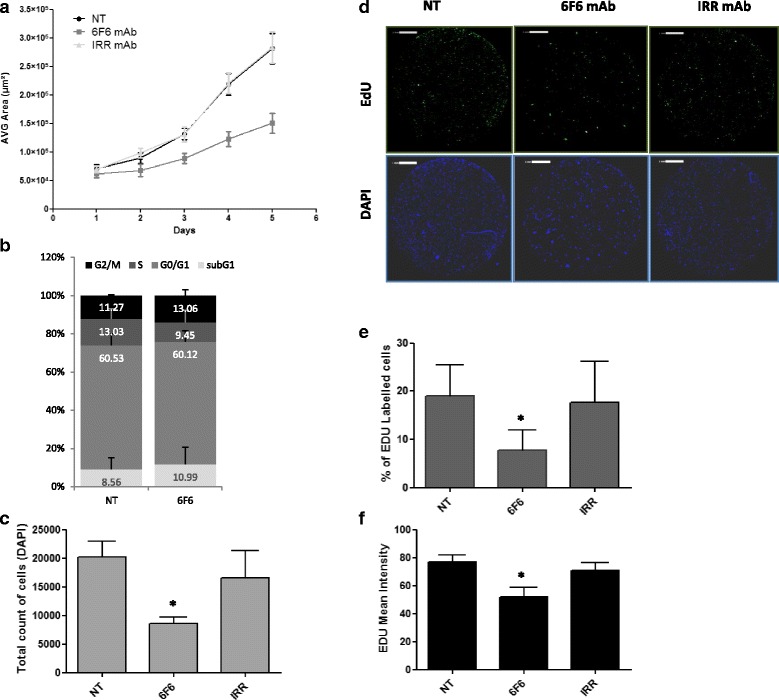
Anti-proliferative effect of the 6F6 mAb on Difi spheroids. **a** The Celigo™ imaging cytometer was used to monitor growth and measure the average (AVG) spheroid area over 5 days. Bright-field images of Difi spheroids incubated or not (NT) with the 6F6 mAb or the irrelevant (IRR) mAb (negative control) were acquired every day. **b** Cell cycle distribution in DiFi spheroids was assessed by quantifying propidium iodide staining with a FC500 flow cytometer after incubation or not (NT) with the 6F6 mAb for 5 days. **c** Quantification of the number of DAPI-positive cells in Difi spheroids incubated or not (NT) with the 6F6 or the irrelevant (IRR) mAb at day 5. **d** Zoomed images at day 5 of spheroids incubated or not (NT) with the 6F6 or the irrelevant (IRR) mAb and then stained with EdU (*green*) and DAPI (*blue*) for the last 24 h. **e** After 24 h EdU incorporation, quantification of EdU-positive cells and **f** measurement of EdU fluorescence intensity at day 5 in spheroids incubated or not (NT) with the 6F6 or the irrelevant (IRR) mAb. * = *p* < 0.05 (paired *t*-test)

To determine whether 6F6 inhibitory effects involved promotion of cell death or inhibition of cell proliferation, the growth of Difi spheroids was monitored over time. After 5 days of culture in the presence of the 6F6 mAb, the spheroid area was significantly reduced (by more than two-fold) compared with untreated cultures or incubated with the irrelevant antibody (Fig. 5a). Then, quantification of propidium iodide staining showed a similar cell cycle distribution in treated and untreated spheroids, although the S phase fraction was slightly reduced in 6F6-treated spheroids (from 13.3 to 9.45%) (Fig. 5b). Thus, the percentage of cells in S phase was evaluated by quantifying EdU incorporation between day 4 and 5 of culture. In DiFi spheroids incubated with the 6 F6 mAb, the number of DAPI-labeled cells (total cell count) was significantly reduced (*p* = 0.02 vs untreated cultures) (Fig. 5c, d) as well as the percentage of EdU-positive cells (19% in untreated and 7.7% in 6 F6-treated cultures; *p =* 0.05) and EdU mean intensity (*p* = 0.01 vs untreated cultures) (Fig. 5d, e, f). The irrelevant antibody did not have any effect.

Quantification of caspase-3 activation (a marker of apoptosis) in live Difi spheroids at day 5 did not show any significant difference in caspase-3 activation between negative controls and spheroids incubated with the 6F6 mAb (Additional file 2: Figure S4; Additional file 3). As a positive control, incubation with cetuximab significantly increased caspase-3 activation in spheroids compared with the other conditions (*p* = 0.05), as already described [32].

Finally, the 6F6 mAb reduced migration of the CDN1-positive SW620 and Caco2 cell lines (43 ± 9% reduction in Caco2 cells compared with untreated cells or incubated with the irrelevant antibody). Similarly, in wound healing assays, migration of SW620 cells was inhibited by the 6F6 mAb (Additional file 2: Figure S5; Additional file 3).

### The anti-CLDN1 monoclonal antibody delays CRC cell xenograft growth and metastasis formation

**Fig. 6:**
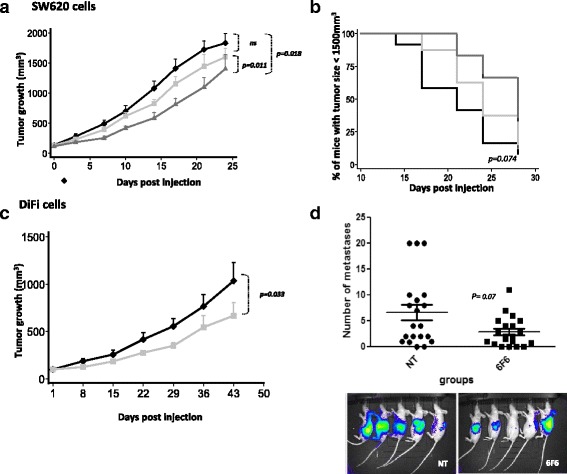
Therapeutic effects of the 6F6 mAb in vivo. **a**, Effect of the 6F6 mAb on the growth of SW620 cell xenografts in athymic nude mice. Mice were treated or not (*black line*) with 15 mg/kg twice (*light gray line*) or three times per week (*dark gray line*) when tumors reached 100 mm^3^ (*n* = 8 animals per group). **b**, Adapted Kaplan-Meier curves using the time taken to reach a tumor volume of 1500 mm^3^ in untreated mice (*black solid line*) and in animals treated with the 6F6 mAb twice (*gray solid line*) or three times per week (*gray dotted line*) (log-rank test). **c**, Effect of the 6F6 mAb on DiFi cell xenografts. Mice received (*gray line*) or not (*dark line*) 15 mg/kg of the 6F6 mAb twice per week. **d**, Effect of the 6F6C mAb on liver metastasis formation. Mice were treated or not with 15 mg/kg of 6F6 three times per week after splenic injection of SW620-LUC cells. *Top*: number of liver metastases in 6F6-treated and untreated (NT) mice (*n* = 20/group). C test. *Bottom*: representative in vivo luminescence images at week 5 after surgery. Five mice are shown for each group

To evaluate the therapeutic potential of the anti-CLDN1 6F6 mAb, athymic nude mice were xenografted with SW620 cells (*KRAS*-mutated) and treated with 0.9% NaCl or the 6F6 mAb. Measurement of the tumor volume over time showed that tumor growth was significantly reduced in the two 6F6 mAb-treated groups (*p* = 0.018) compared with controls. This effect was significantly stronger (*p* = 0.011) in the group treated three times per week than in the group that received the mAb only twice per week (Fig. 6a). The median time needed for the tumor to reach the volume of 1500 mm^3^ was longer in the group treated three times/week compared with controls (28 days vs 21 days, respectively; *p* = 0.07) (Fig. 6b). The inhibitory effect of the 6F6 mAb on tumor growth was confirmed in mice xenografted with DiFi CRC cells (wild type *KRAS*) that overexpress CLDN1 (*p* = 0.03) (Fig. 6c).

Finally, analysis of the effects of the 6 F6 mAb on the formation of liver metastases showed that SW620 cells metastasized to the liver, as previously reported [14], but the number of metastases per liver was lower in the mAb-treated group than in controls (*p* = 0.07) (Fig. 6d).

## Discussion

In this study, we assessed CLDN1 as a potential therapeutic target in CRC. We found that CLDN1 is mainly localized at the cell membrane of CRC cells and described, for the first time, CLDN1 expression in the different CRC molecular subtypes. We also found that CLDN1 expression has a prognostic value in the C3 and C5 Marisa’s subtypes. Using a new mAb (6F6) that specifically recognizes the extracellular part of human CLDN1, we demonstrated that CLDN1 targeting reduces CRC xenograft growth and liver metastasis formation. Finally, we showed that 6F6 activity is mediated through inhibition of cell proliferation.

CLDN1 membrane overexpression in all tested primary tumor samples from patients with mCRC supports the hypothesis that CLDN1 could represent a target for antibody-based therapy. Analysis of CLDN1 expression in the new CRC molecular subtypes [23–25] and the recent consensus subtypes [26] highlighted important variations among the different CRC classes, in agreement with CRC heterogeneity. Specifically, *CLDN1* expression is significantly higher in CRC subtypes associated with marked WNT signaling activation, such as the Marisa’s C5, Sadanandam’s TA and CMS2 consensus subtypes. These findings are in accordance with the already described involvement of CLDN1 in WNT signaling [13, 14, 16]. Moreover, we demonstrated for the first time that *CLDN1* expression level could be used for outcome prediction in patients with mCRC and that this prognostic value is dependent on the molecular subtype. CLDN1 could become a marker to guide the choice of targeted therapy in mCRC. Indeed, CRCs belonging to the Marisa’s C5 and C3 subtypes could be targeted with an anti-CLDN1 mAb, particularly the C3 subtype because it is enriched in CRCs from patients with mutated *KRAS* who are not eligible to anti-EGFR targeted therapy [33]. Moreover, the in vivo anti-tumor effect (tumor growth delay and survival improvement) of the 6F6 mAb in mice xenografted with CLDN-positive CRC cells seems to be independent of *KRAS* mutational status.

In vitro, the 6 F6 mAb negatively influences cell growth when CRC cells are grown in a 3D culture system. Growing evidence indicates that cancer cells cultured in 3D spheroids are closer to in vivo models than cell cultured in 2D systems and that they might better predict the in vivo outcome [34]. Indeed, CLDN1 is critical for maintaining cell growth in 3D, but does not affect cell growth in monolayer cultures [35]. Our findings indicate that in 3D culture, the 6F6 mAb has a cytostatic effect on tumor cells. As both treated and untreated spheroids showed similar cell cycle distribution, this cytostatic effect reflects a global slowing down of cell cycle progression and not an arrest in a particular phase.

In the intrasplenic model of liver metastases, which results in the rapid liver colonization by tumor cells with extremely aggressive growth [36], treatment with the 6F6 mAb decreased the number of liver metastases. These results are supported by the in vitro finding that the 6F6 mAb decreases the number of colonies and reduces cell survival in a large panel of CLDN1-positive cancer cell lines. Moreover, the 6F6 mAb affected the migration capacity of all tested CLDN1-positive CRC cell lines. In agreement, siRNA-mediated *CLDN1* knock-down in metastatic CRC cells inhibits migration [14]. Conversely, CLDN1 overexpression increases cell motility [37]. Our anti-CLDN1 mAb, by affecting tumor cell migration, could play an important role in the control of cancer cell invasiveness.

The efficacy of the 6F6 mAb could be improved by increasing its Fc-dependent properties. Indeed, the 6 F6 mAb is an IgG3 and, therefore, only binds with low affinity to FcgammaRI [38] that mediates antibody-dependent cell-mediated cytotoxicity. Another approach to enhance 6 F6 mAb efficacy would be to associate a cytotoxic effector agent in order to create an antibody–drug conjugate [39]. Before that, its toxicity in healthy tissues must be evaluated, although a recent study excluded major toxicity or side effects induced by another anti-CLDN1 mAb in mice [40].

## Conclusions

This study demonstrated that CLDN1 could be a new potential therapeutic target in CRC and that CLDN1 targeting with a specific antibody has anti-tumor effects in vivo and in vitro. Moreover, analysis of CLDN1 expression in primary tumor samples from patients with mCRC allowed the identification of two CRC molecular subclasses (C3 and C5) that could benefit from CLDN1-targeting therapies. Finally, this work provides the proof of concept for the development of new therapeutic strategies against tight junction proteins in CRC.

## Additional files

Additional file 1: Table S1. Distribution of patients with mCRC according to the tumor molecular subtype. (DOCX 33 kb)

Additional file 2: Figure S1. *CLDN1* gene (222549_at.) expression. **a**, in 17 normal colorectal mucosa (NM), 20 primary tumor (PT) samples and 19 hepatic metastases (HM); *** = *p* < 0.0001 (Kruskall Wallis/Dunn’s test). **b**, Ratio between *CLDN1* expression in PT and *CLDN1* expression in NM for the 15 paired NM and PT samples from patients with mCRC. Data from the Affymetrix GeneChip Human Genome U133 Array Set (GSE 62322). **Figure S2.** The 6F6 mAb is specific for CLDN1. **a**, Reactivity of the hybridoma supernatant 6F6 against CLDN1. Western blotting of protein extracts from SW480 cells stably transfected with CLDN1 and from SW620 cells transduced with shLUC (control) or ShCLDN1. FACS histograms show the binding of the hybridoma supernatant to CLDN1-positive cell lines (SW480-CLDN1 and SW620shLUC) (■), negative control (-----), CLDN1-negative cell lines (―). **b**, Immunofluorescence experiments in cells that express CLDN1 (SW480-CLDN1) or transfected with empty vector (SW480-pcDNA) using the 6 F6 mAb as primary antibody (green). Images were recorded using a 63X NA objective on a Leica inverted microscope. **c**, Surface plasmon resonance measurements of the interaction of 6F6 or of an irrelevant mAb (Irr) with membrane extracts from SW620 cells that express CLDN1. **d**, Cross-reactivity analysis of the 6F6 mAb towards other CLDN proteins. Top: The expression of the various CLDN proteins (as indicated) in cell lysates from parental or CLDN-transfected SW480 cells was tested by western blotting using the relevant antibodies; Bottom: FACS histograms of 6 F6 binding (10 μg/mL) to parental or CLDN-transfected SW480 cells. Gray, 6 F6 mAb; dotted line, no antibody; black line, irrelevant mAb. **Figure S3.** CLDN1 is expressed in various cancer cell lines **a**, FACS histograms of the 6F6 mAb binding (gray histogram) to different cancer cell lines (pancreatic cancer: PANC-1, BXPC-3; ovarian cancer: SKOV-3, IGROV-1; hepatocarcinoma: HUH7). **b**, Quantification of total CLDN1 expression in the cell lines used in **a** by western blotting using the anti-CLDN1 polyclonal antibody JAY-8. **c**, CLDN1 mRNA expression in cell lines from the Cancer Cell Line Encyclopedia (http://www.broadinstitute.org/ccle). **Figure S4.** Detection of apoptosis in Difi spheroids using the Celigo™ imaging system and the NucView™ 488 cell membrane-permeable fluorogenic caspase-3 substrate. Difi cells were seeded at a density of 10^4^/ml in FluoroBrite™ DMEM supplemented with 10% fetal bovine serum and incubated or not (NT) with 100 μg/ml of the 6 F6 mAb, the anti-EGFR cetuximab (cetux) or an irrelevant mAb (IRR). The caspase-3 substrate was added (5 μM) at the same time. Images were acquired at day 5. The bright-field and caspase 3 (green) images were merged (top panels) and the histogram (lower panel) represents the mean fluorescence intensity; * = *p* < 0.05 (*t*-test). **Figure S5.** Effects of the 6F6 mAb on cancer cell migration in vitro. **a**, Wound healing assay: confluent SW620 cell monolayers were scratched and then grown in the presence or not (NT) of 100 μg/ml of the 6 F6 mAb or irrelevant antibody (IRR). Images were captured at day 0 (D0) and day 5 (D5) after wounding. **b**, Cell migration assay in Boyden chambers. Caco2 cells were pre-incubated or not (NT) with 100 μg/ml of 6F6 or irrelevant (IRR) mAb. Data used for statistical analysis were from at least three independent experiments; **p < 0.01 (Kruskall Wallis/Dunn’s test). (PPTX 2370 kb)

Additional file 3: Supplementary methods: Flow cytometry experiments. Immunofluorescence studies. Surface plasmon resonance measurements. Cell migration assays. Apoptosis assay. (DOCX 37 kb)
