# Supplementary material for: Antibody targeting of claudin-1 as a potential colorectal cancer therapy
Source: J Exp Clin Cancer Res. 2017 Jun 28;36:89. doi: 10.1186/s13046-017-0558-5 (PMC5490170; doi:10.1186/s13046-017-0558-5)
Supplement: Supplementary file 1 — Distribution of patients with mCRC according to the tumor molecular subtype. (DOCX 33 kb) [file 13046_2017_558_MOESM1_ESM.docx]

**Table S1: Distribution of patients with mCRC according to the tumor molecular subtype**

| **Subtypes** | **N = 143** | **%** |
| --- | --- | --- |
| **De Sousa** (n=139) |  |  |
| CCS1 | 76 | 54.7% |
| CCS2 | 19 | 13.7% |
| CCS3 | 44 | 31.7% |
| Missing | 4 |  |
| **Sadanandam** (n=129) |  |  |
| Enterocyte | 37 | 28.7 |
| Goblet-like | 15 | 11.6 |
| Inflammatory | 16 | 12.4 |
| Stem-like | 37 | 28.7 |
| TA | 24 | 18.6 |
| Missing | 14 |  |
| **Marisa** (n=138) |  |  |
| C1 | 27 | 19.6 |
| C2 | 17 | 12.3 |
| C3 | 19 | 13.8 |
| C4 | 20 | 14.5 |
| C5 | 36 | 26.1 |
| C6 | 19 | 13.8 |
| Missing | 5 |  |
| **Consensus** (n= 104) |  |  |
| CMS1 | 15 | 14.4 |
| CMS2 | 29 | 27.9 |
| CMS3 | 24 | 23.1 |
| CMS4 | 36 | 34.6 |
| Missing | 39 |  |
