# Supplementary material for: Antibody targeting of claudin-1 as a potential colorectal cancer therapy
Source: J Exp Clin Cancer Res. 2017 Jun 28;36:89. doi: 10.1186/s13046-017-0558-5 (PMC5490170; doi:10.1186/s13046-017-0558-5)
Supplement: Supplementary file 2 — CLDN1 gene (222549_at.) expression. a, in 17 normal colorectal mucosa (NM), 20 primary tumor (PT) samples and 19 hepatic metastases (HM); *** = p < 0.0001 (Kruskall Wallis/Dunn’s test). b, Ratio between CLDN1 expression in PT and CLDN1 expression in NM for the 15 paired NM and PT samples from patients with mCRC. Data from the Affymetrix GeneChip Human Genome U133 Array Set (GSE 62322). Figure S2. The 6F6 mAb is specific for CLDN1. a, Reactivity of the hybridoma supernatant 6F6 against CLDN1. Western blotting of protein extracts from SW480 cells stably transfected with CLDN1 and from SW620 cells transduced with shLUC (control) or ShCLDN1. FACS histograms show the binding of the hybridoma supernatant to CLDN1-positive cell lines (SW480-CLDN1 and SW620shLUC) (■), negative control (-----), CLDN1-negative cell lines (―). b, Immunofluorescence experiments in cells that express CLDN1 (SW480-CLDN1) or transfected with empty vector (SW480-pcDNA) using the 6 F6 mAb as primary antibody (green). Images were recorded using a 63X NA objective on a Leica inverted microscope. c, Surface plasmon resonance measurements of the interaction of 6F6 or of an irrelevant mAb (Irr) with membrane extracts from SW620 cells that express CLDN1. d, Cross-reactivity analysis of the 6F6 mAb towards other CLDN proteins. Top: The expression of the various CLDN proteins (as indicated) in cell lysates from parental or CLDN-transfected SW480 cells was tested by western blotting using the relevant antibodies; Bottom: FACS histograms of 6 F6 binding (10 μg/mL) to parental or CLDN-transfected SW480 cells. Gray, 6 F6 mAb; dotted line, no antibody; black line, irrelevant mAb. Figure S3. CLDN1 is expressed in various cancer cell lines a, FACS histograms of the 6F6 mAb binding (gray histogram) to different cancer cell lines (pancreatic cancer: PANC-1, BXPC-3; ovarian cancer: SKOV-3, IGROV-1; hepatocarcinoma: HUH7). b, Quantification of total CLDN1 expression in the cell lines used in a by western blotting usin [file 13046_2017_558_MOESM2_ESM.pptx]

## Slide 1
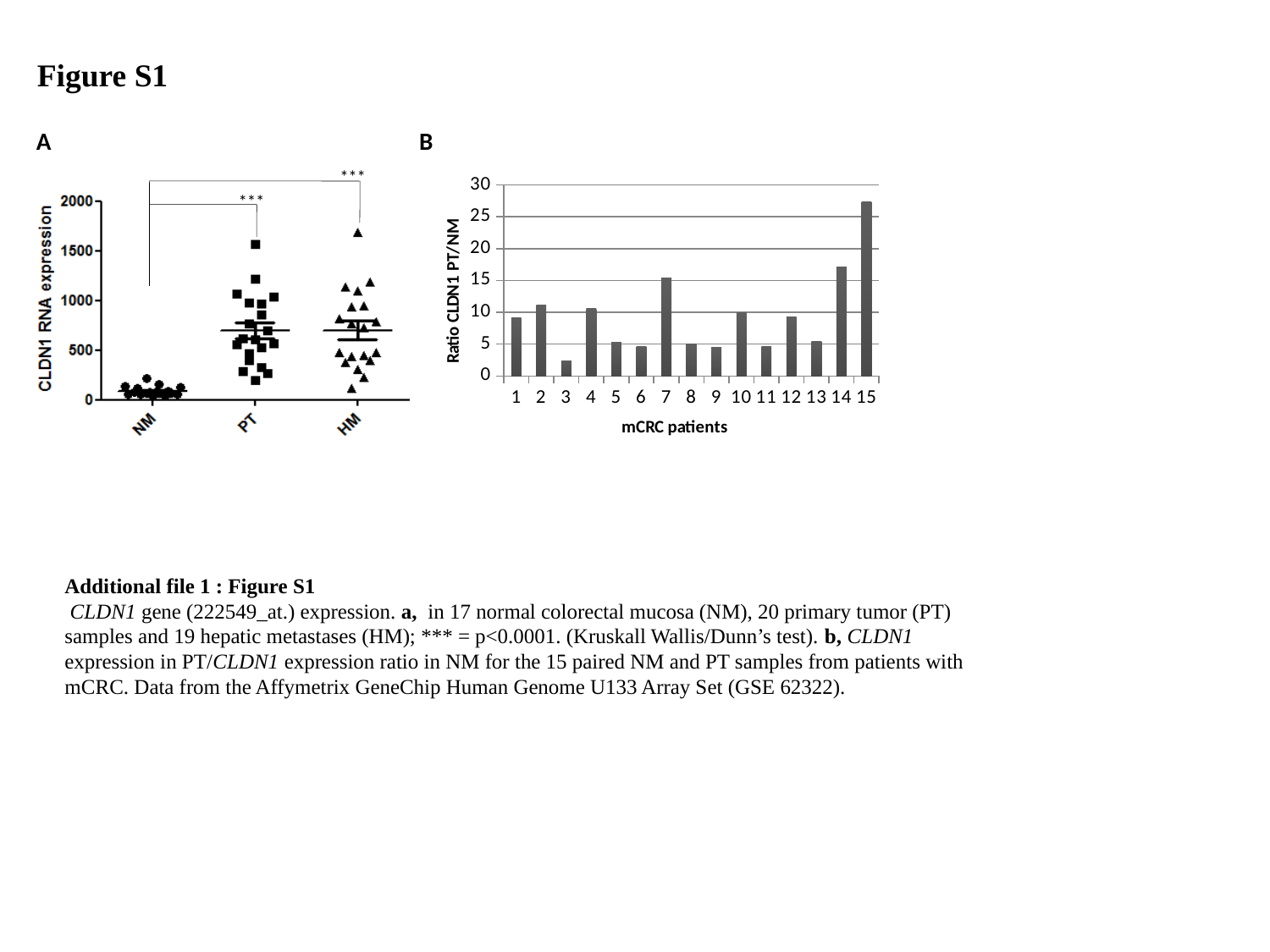

Figure S1
A
B
### Chart
| Category | Ratio PT/NM |
|---|---| ***
 ***
Additional file 1 : Figure S1
 CLDN1 gene (222549_at.) expression. a, in 17 normal colorectal mucosa (NM), 20 primary tumor (PT) samples and 19 hepatic metastases (HM); *** = p<0.0001. (Kruskall Wallis/Dunn’s test). b, CLDN1 expression in PT/CLDN1 expression ratio in NM for the 15 paired NM and PT samples from patients with mCRC. Data from the Affymetrix GeneChip Human Genome U133 Array Set (GSE 62322).

## Slide 2
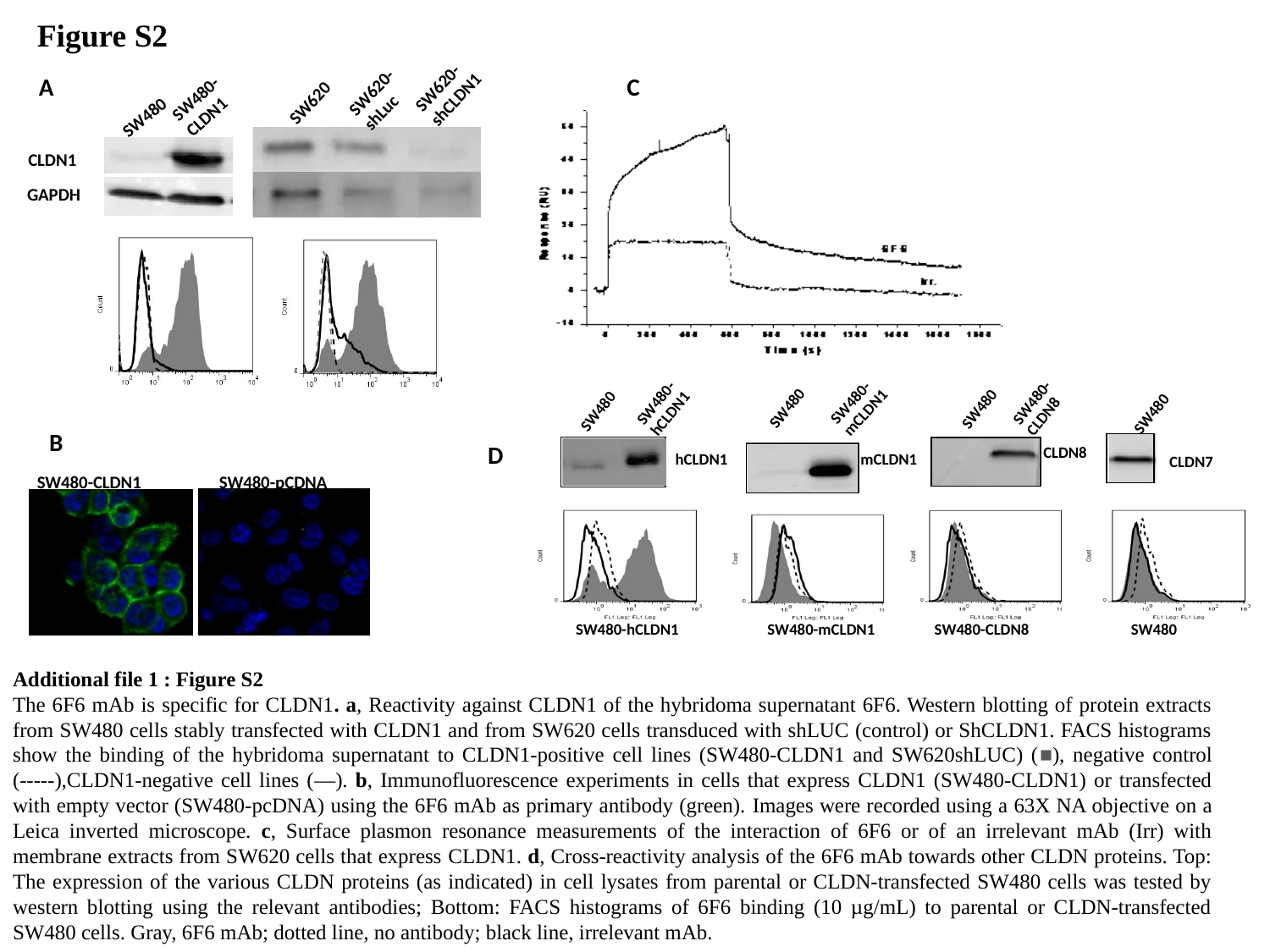

SW620-shCLDN1
SW620-shLuc
SW480-CLDN1
SW620
SW480
CLDN1
GAPDH
Figure S2
A
C
SW480-hCLDN1
SW480
hCLDN1
SW480-CLDN8
SW480
CLDN8
SW480
CLDN7
SW480-mCLDN1
SW480
mCLDN1
SW480-CLDN8
SW480
SW480-hCLDN1
SW480-mCLDN1
B
D
SW480-CLDN1
SW480-pCDNA
Additional file 1 : Figure S2
The 6F6 mAb is specific for CLDN1. a, Reactivity against CLDN1 of the hybridoma supernatant 6F6. Western blotting of protein extracts from SW480 cells stably transfected with CLDN1 and from SW620 cells transduced with shLUC (control) or ShCLDN1. FACS histograms show the binding of the hybridoma supernatant to CLDN1-positive cell lines (SW480-CLDN1 and SW620shLUC) (■), negative control (-----),CLDN1-negative cell lines (―). b, Immunofluorescence experiments in cells that express CLDN1 (SW480-CLDN1) or transfected with empty vector (SW480-pcDNA) using the 6F6 mAb as primary antibody (green). Images were recorded using a 63X NA objective on a Leica inverted microscope. c, Surface plasmon resonance measurements of the interaction of 6F6 or of an irrelevant mAb (Irr) with membrane extracts from SW620 cells that express CLDN1. d, Cross-reactivity analysis of the 6F6 mAb towards other CLDN proteins. Top: The expression of the various CLDN proteins (as indicated) in cell lysates from parental or CLDN-transfected SW480 cells was tested by western blotting using the relevant antibodies; Bottom: FACS histograms of 6F6 binding (10 µg/mL) to parental or CLDN-transfected SW480 cells. Gray, 6F6 mAb; dotted line, no antibody; black line, irrelevant mAb.

## Slide 3
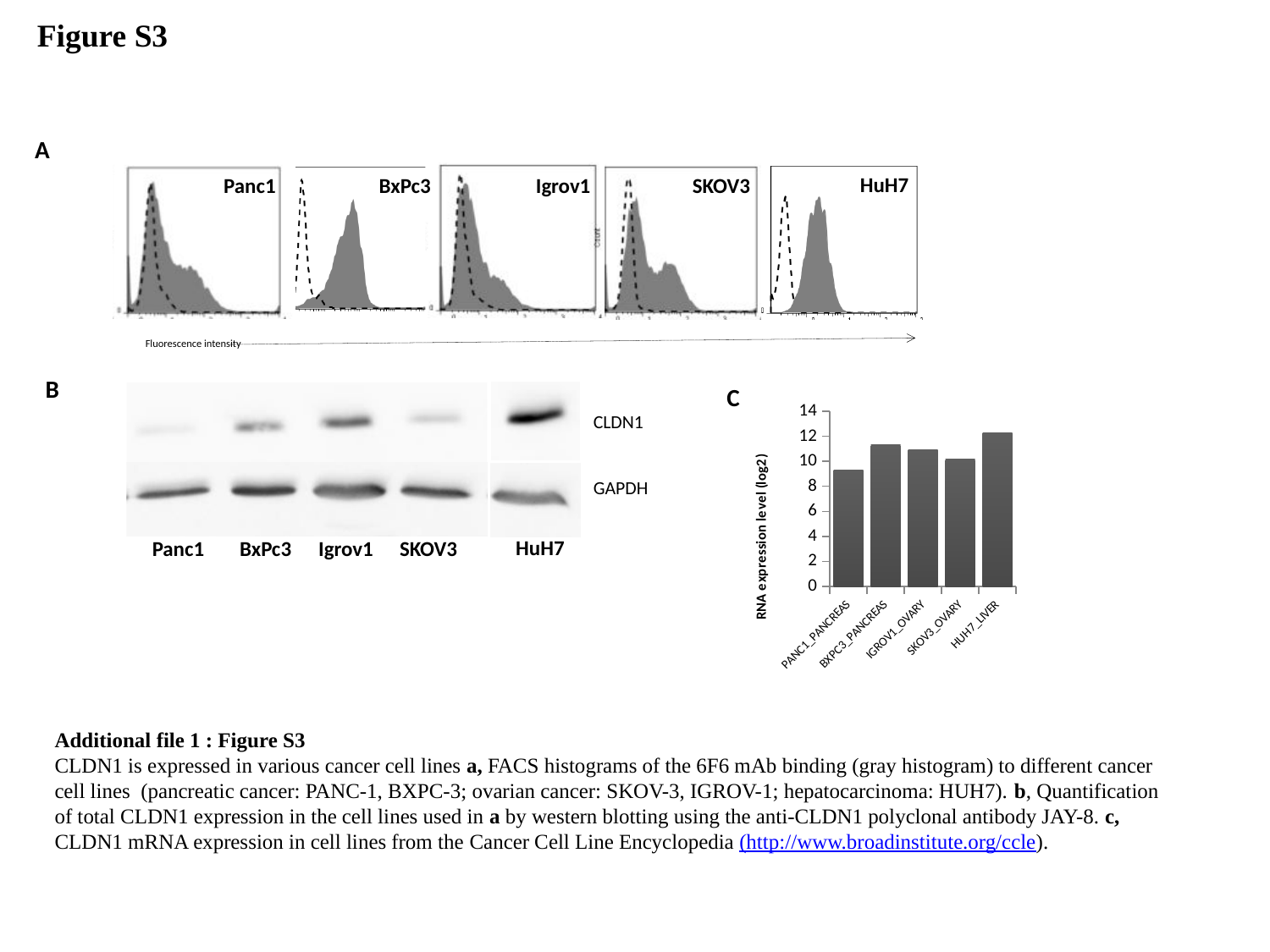

Figure S3
A
Fluorescence intensity
HuH7
Panc1
BxPc3
Igrov1
SKOV3
B
C
HuH7
Panc1
BxPc3
Igrov1
SKOV3
CLDN1
GAPDH
### Chart
| Category | RNA expression level (log2) |
|---|---|
| PANC1_PANCREAS | 9.271697000000001 |
| BXPC3_PANCREAS | 11.331620000000001 |
| IGROV1_OVARY | 10.91407 |
| SKOV3_OVARY | 10.19054 |
| HUH7_LIVER | 12.27607 |
Additional file 1 : Figure S3
CLDN1 is expressed in various cancer cell lines a, FACS histograms of the 6F6 mAb binding (gray histogram) to different cancer cell lines (pancreatic cancer: PANC-1, BXPC-3; ovarian cancer: SKOV-3, IGROV-1; hepatocarcinoma: HUH7). b, Quantification of total CLDN1 expression in the cell lines used in a by western blotting using the anti-CLDN1 polyclonal antibody JAY-8. c, CLDN1 mRNA expression in cell lines from the Cancer Cell Line Encyclopedia (http://www.broadinstitute.org/ccle).

## Slide 4
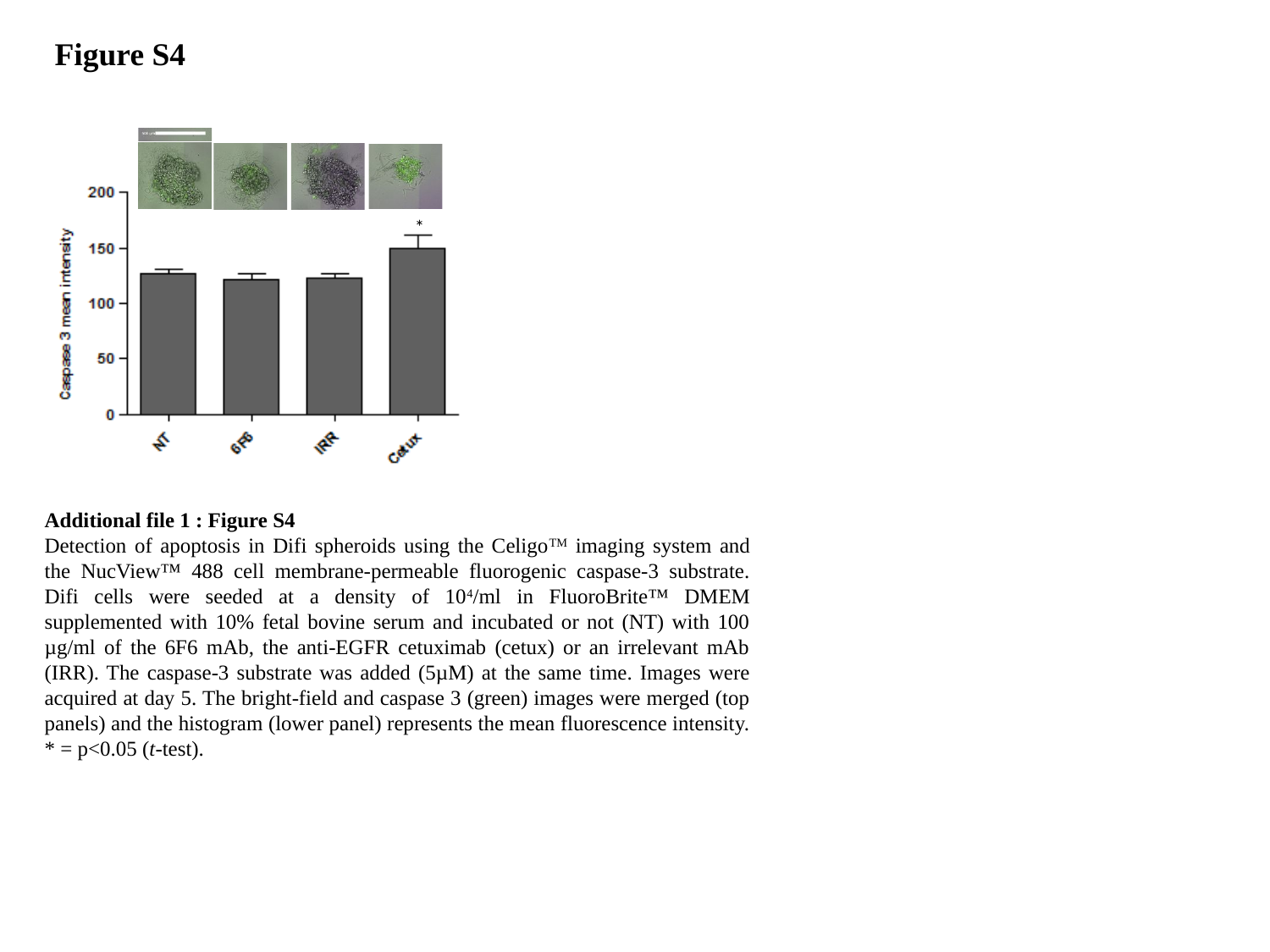

Figure S4
*
Additional file 1 : Figure S4
Detection of apoptosis in Difi spheroids using the CeligoTM imaging system and the NucView™ 488 cell membrane-permeable fluorogenic caspase-3 substrate. Difi cells were seeded at a density of 104/ml in FluoroBrite™ DMEM supplemented with 10% fetal bovine serum and incubated or not (NT) with 100 µg/ml of the 6F6 mAb, the anti-EGFR cetuximab (cetux) or an irrelevant mAb (IRR). The caspase-3 substrate was added (5µM) at the same time. Images were acquired at day 5. The bright-field and caspase 3 (green) images were merged (top panels) and the histogram (lower panel) represents the mean fluorescence intensity. * = p<0.05 (t-test).

## Slide 5
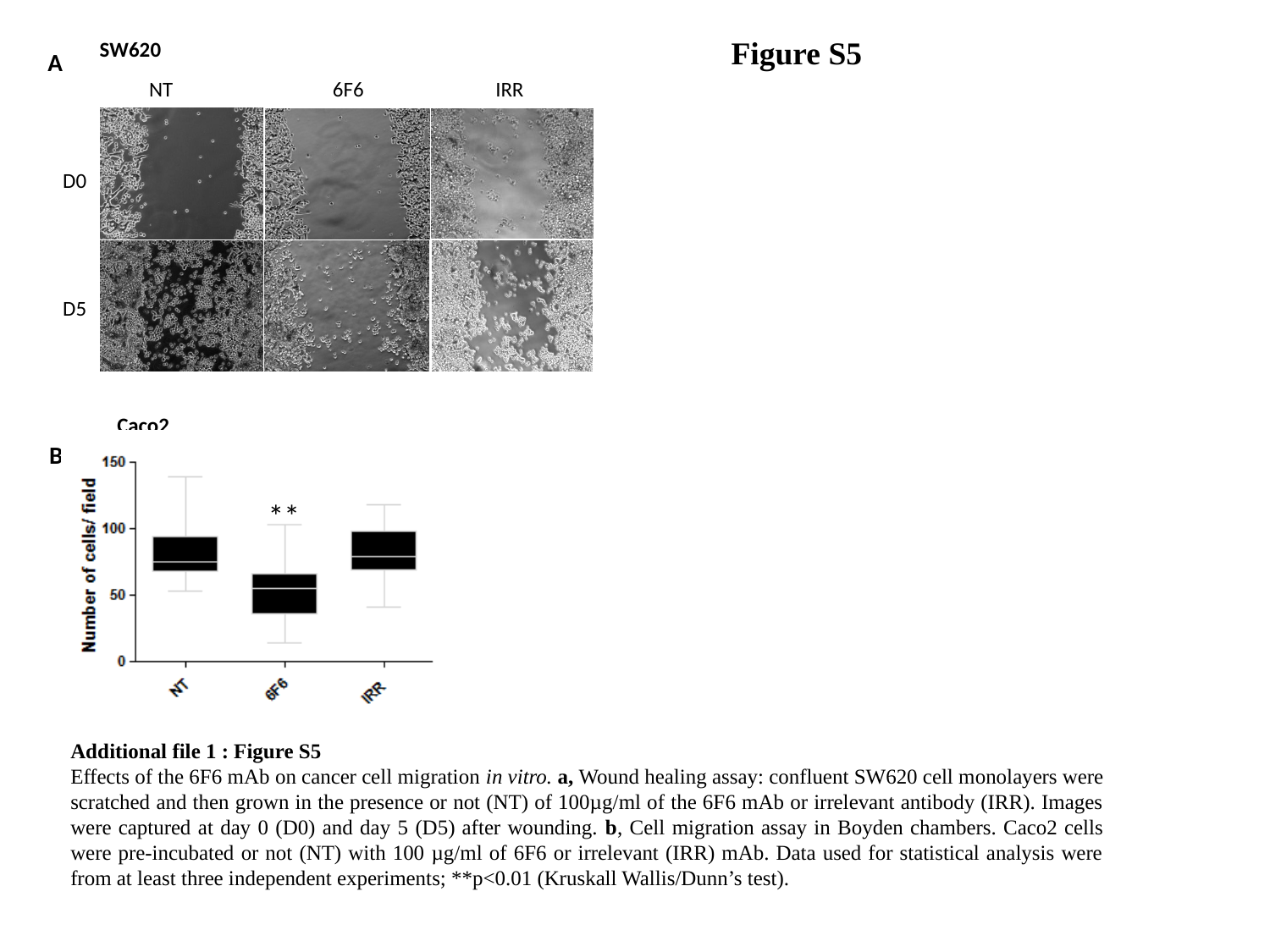

Figure S5
SW620
A
NT
6F6
IRR
D0
D5
Caco2
**
B
Additional file 1 : Figure S5
Effects of the 6F6 mAb on cancer cell migration in vitro. a, Wound healing assay: confluent SW620 cell monolayers were scratched and then grown in the presence or not (NT) of 100µg/ml of the 6F6 mAb or irrelevant antibody (IRR). Images were captured at day 0 (D0) and day 5 (D5) after wounding. b, Cell migration assay in Boyden chambers. Caco2 cells were pre-incubated or not (NT) with 100 µg/ml of 6F6 or irrelevant (IRR) mAb. Data used for statistical analysis were from at least three independent experiments; **p<0.01 (Kruskall Wallis/Dunn’s test).
