# Supplementary material for: Antibody targeting of claudin-1 as a potential colorectal cancer therapy
Source: J Exp Clin Cancer Res. 2017 Jun 28;36:89. doi: 10.1186/s13046-017-0558-5 (PMC5490170; doi:10.1186/s13046-017-0558-5)
Supplement: Supplementary file 3 — Supplementary methods: Flow cytometry experiments. Immunofluorescence studies. Surface plasmon resonance measurements. Cell migration assays. Apoptosis assay. (DOCX 37 kb) [file 13046_2017_558_MOESM3_ESM.docx]

**Additional file 3: Supplementary methods**

**Flow cytometry experiments**

Hybridoma supernatants and mAb binding were assessed using a fluorescence-activated cell sorter (Quanta apparatus, Beckman Coulter). Cells were seeded in 25cm^2^ flasks (2 x 10^5^ cells/flask). After 48 hours, one million cells were pelleted, washed with PBS/1% BSA and incubated or not with hybridoma supernatants or mAbs, on ice for 1h. After washing, an AlexiaFluor488-conjugated-goat anti-mouse monoclonal antibody (1:500 dilution; Invitrogen) was added on ice for 45min to detect interactions with the primary antibodies. Incubation of cells with only the secondary antibody was used for background measurements (negative control).

**Immunofluorescence studies**

Cells were plated on 12 mm glass coverslips in culture dishes. One day after plating, cells were fixed with 4% paraformaldehyde/PBS at room temperature for 10 min and blocked with PBS containing 5% BSA at 37°C for 30 min. Cells were then incubated with 10 µg/ml of the 6F6 mAb for 1h and then with a FITC-conjugated goat anti-mouse IgG (H+L) (Invitrogen). Nuclei were stained with DAPI. Cells were mounted in Moviol, and images acquired using a 63X NA objective on a Leica inverted microscope.

**Surface plasmon resonance measurements**

Binding of the 6F6 mAb and irrelevant antibodies was assessed using a Biacore 3000 instrument at 25°C in PBS. Cell membrane extracts were immobilized at 2600 RU on the HPA sensor chip surface according to the manufacturer’s specifications. Antibodies were injected at 660nM over the immobilized LPS at a flow rate of 2µL/min during 10min followed by a 600s dissociation step with PBS running buffer. To make cell membrane extracts, SW480-CLDN1 cells were washed three times with cold PBS and incubated with 1ml of 10mM Tris pH7.2 for 30 minutes. Then, they were scraped and sonicated four times for 5 seconds. Protein extracts were centrifuged at 7000g for 15min and supernatants ultra-centrifuged at 200 000g for 15min. Pellets were sonicated and resuspended in PBS. Protein concentration was evaluated using the BCA protein assay reagent (Pierce).

**Cell migration assays**

Cell migration was studied in Boyden chambers or by wound healing. Briefly, Caco2 cells were incubated with 100 µg/ml of the 6F6 mAb for two hours and then they were trypsinized, washed three times with serum-free medium and 100 000 cells were added onto Transwell inserts (pore size: 8µm; BD Falcon^®^ HTS Fluoroblok^TM^ Inserts). The lower compartment was filled with medium supplemented with 10% FBS. After 21h incubation, migrated cells were stained with 4µg/ml calcein (Sigma-Aldrich 17783-AM) for 1h. The number of migrated cells was counted in 12 different fields using the ImageJ software.

SW620 cells were seeded in 12-well plates and grown at 37°C in RPMI medium with 10% FBS. At 90% confluence, a wound was generated by scratching each monolayer with a pipette tip. Cells were then incubated in medium with 100 μg/ml of the 6F6 mAb, or not. Images were captured at day 0 and day 5 after wounding with a Leica microscope.

**Apoptosis assay**

Detection of apoptosis in Difi spheroids was done using the NucView™ 488 cell membrane-permeable fluorogenic caspase-3 substrate designed for detecting caspase-3 activity within live cells in real time. Difi spheroids treated or not with 100 µg/ml of the 6F6 mAb, the anti-EGFR cetuximab or an irrelevant mAb were then incubated with caspase-3 substrate. Image acquisition (fluorescence and bright-field) was made by Celigo™ imaging cytometer using Celigo software Expression Analysis: "Target 1+2".
